# Patterns of loco-regional progression and patient outcomes after definitive-dose radiation therapy for anaplastic thyroid cancer

**DOI:** 10.1016/j.radonc.2024.110602

**Published:** 2024-11-01

**Authors:** Julianna K. Bronk, Alexander Augustyn, Abdallah S.R. Mohamed, C. David Fuller, Adam S. Garden, Amy C. Moreno, Anna Lee, William H. Morrison, Jack Phan, Jay P. Reddy, David I. Rosenthal, Michael T. Spiotto, Steven J. Frank, Ramona Dadu, Naifa Busaidy, Mark Zafereo, Jennifer R. Wang, Anastasios Maniakas, Renata Ferrarotto, Priyanka C. Iyer, Maria E. Cabanillas, G. Brandon Gunn

**Affiliations:** a Department of Radiation Oncology, University of Texas MD Anderson Cancer Center, Houston, TX, United States; b Community Health Network Hospital, Indianapolis, IN, United States; c Department of Endocrine Neoplasia and Hormonal Disorders, University of Texas MD Anderson Cancer Center, Houston, TX, United States; d Department of Head and Neck Surgery, University of Texas MD Anderson Cancer Center, Houston, TX, United States; e Department of Thoracic-Head and Neck Medical Oncology, University of Texas MD Anderson Cancer Center, Houston, TX, United States

**Keywords:** Anaplastic thyroid cancer, Definitive intensity modulated radiation therapy, Patterns of failure

## Abstract

**Background:**

The aim of this study is to characterize the patterns of loco-regional progression (LRP) and outcomes after definitive-dose intensity modulated radiation therapy (IMRT) for anaplastic thyroid cancer (ATC) with macroscopic neck disease at the time of IMRT.

**Methods:**

Disease/treatment characteristics and outcomes for patients with unresected or incompletely resected ATC who received IMRT (≥45 Gy) were retrospectively reviewed. For those with LRP after IMRT, progressive/recurrent gross tumor volumes (rGTV) were contoured on diagnostic CTs and co-registered with initial planning CTs using deformable image registration. rGTVs were classified based on established spatial/dosimetric criteria.

**Results:**

Forty patients treated between 2010–2020 formed the cohort. Median IMRT dose was 66 Gy (45–70 Gy); altered fractionation (AF) was used in 24 (60 %). All received concurrent chemotherapy. In addition to areas of gross disease, target volumes (TVs) commonly included: central compartment/upper mediastinum (levels VI/VII), neck levels II-V in an involved, and levels III-IV in an uninvolved lateral neck. Median overall survival was 7.1 m. Median progression free survival was 7.4 m for patients with locoregional disease and 1.8 m for patients with distant metastasis at the time of IMRT. Twenty-one patients (53 %) developed LRP at median of 10.9 m; freedom from LRP at 3 m and 12 m was 71 % (95 %CI 58–87 %) and 47 % (95 %CI 32–68 %). Forty-one individual rGTVs were identified and most occurred within the high dose (HD) TVs: Type A/central HD (n = 29, 71 %) and B/peripheral HD (n = 3, 7 %).

**Conclusions:**

Despite an intensive treatment schedule, including AF and concurrent chemotherapy, classic radioresistant and rapid Type A failures predominated; isolated extraneous dose failures were rare. While these findings support the IMRT and TV delineation strategies described herein, they highlight the importance of identifying novel strategies to further improve LRC for patients with unresectable disease without targetable mutations for contemporary neo-adjuvant strategies.

## Introduction

Anaplastic thyroid cancer (ATC) is one of the most aggressive types of cancer and the leading cause of thyroid cancer deaths worldwide [1]. Historical data has shown that the best outcomes for patients with ATC have been achieved with tri-modality therapy consisting of complete surgical resection, post-operative radiation therapy (RT) and systemic therapy [2–5]. More recently, innovations in systemic therapy applied in the neoadjuvant, adjuvant, and salvage settings show great promise towards improving patient survival and have shifted treatment paradigms particularly in patients with BRAFV600E mutated ATC [6–10]. Indeed, modern updated consensus guidelines now support expedited assessment of BRAFV600E status during the initial workup of patients with suspected ATC and incorporate BRAF-directed therapy and immunotherapy in the treatment of borderline-resectable and unresectable disease both prior to and after surgical resection [11,12]. This approach has demonstrated effectiveness in reducing tumor size, extent of surgery and surgical morbidity translating into improvements in overall and progression free survival [9–11].

However, patients with ATC often initially present with rapidly progressive and life-threatening local disease that necessitates upfront loco-regional control (LRC) for effective palliation and to possibly extend survival. Often ATC is not amenable to complete surgical resection due to significant tumor infiltration or disease extent. In the unresectable setting, RT has been considered as an upfront primary local therapy option with the purpose of tumor stabilization, slowing tumor growth, preventing life threatening complications such as airway/esophageal obstruction. Data from a National Cancer Database analysis examining outcomes in over 1200 patients with ATC showed an overall survival (OS) benefit for patients with unresected disease who received RT to a dose of ≥ 45 Gy [13].

While upfront RT may potentially offer both therapeutic and durable palliative benefit in select patients with unresectable (or incompletely resected) ATC without targetable mutations, there is no consensus on the optimal RT strategy in terms of the RT dose, fractionation or target volume (TV) definition. The extent of RT TVs and intensity of therapy must be carefully balanced against risk of treatment-related toxicities in this patient population given tumor location in the low neck and adjacency if not invasion of central neck structures, balanced further against the potential for limited patient prognosis. Here, we examine the anatomic locations, RT dose-categorized distributions and patterns of loco-regional progression (LRP) in patients with unresected or incompletely resected ATC using a validated deformable image registration platform and classification system [14] to objectively evaluate and potentially improve our intensity-modulated RT (IMRT) and TV strategies for these patients.

## Methods

### Patient selection and chart review

This single institution retrospective study was performed after Institutional Review Board approval. Patients eligible for this study were those treated with IMRT (≥45 Gy) from 2010 to 2020 (IMRT era) to the neck at < anonymous > for unresected ATC or after macroscopically incomplete resection (i.e. gross residual neck disease remaining at the time of IMRT). All pathology had been previously reviewed and confirmed ATC histology by a head and neck-specialized pathologist at our institution. Clinical variables collected included: age, date of diagnosis, therapy regimen including surgery and systemic therapy received, RT details including dose, fractionation, and TVs. The presence, location and volume of gross disease (initial gross tumor volumes [iGTVs]) at the time of IMRT was delineated and quantified by a head and neck-specialized radiation oncologist (GBG).

### Recurrent gross tumor volume delineation

**Fig. 1.**
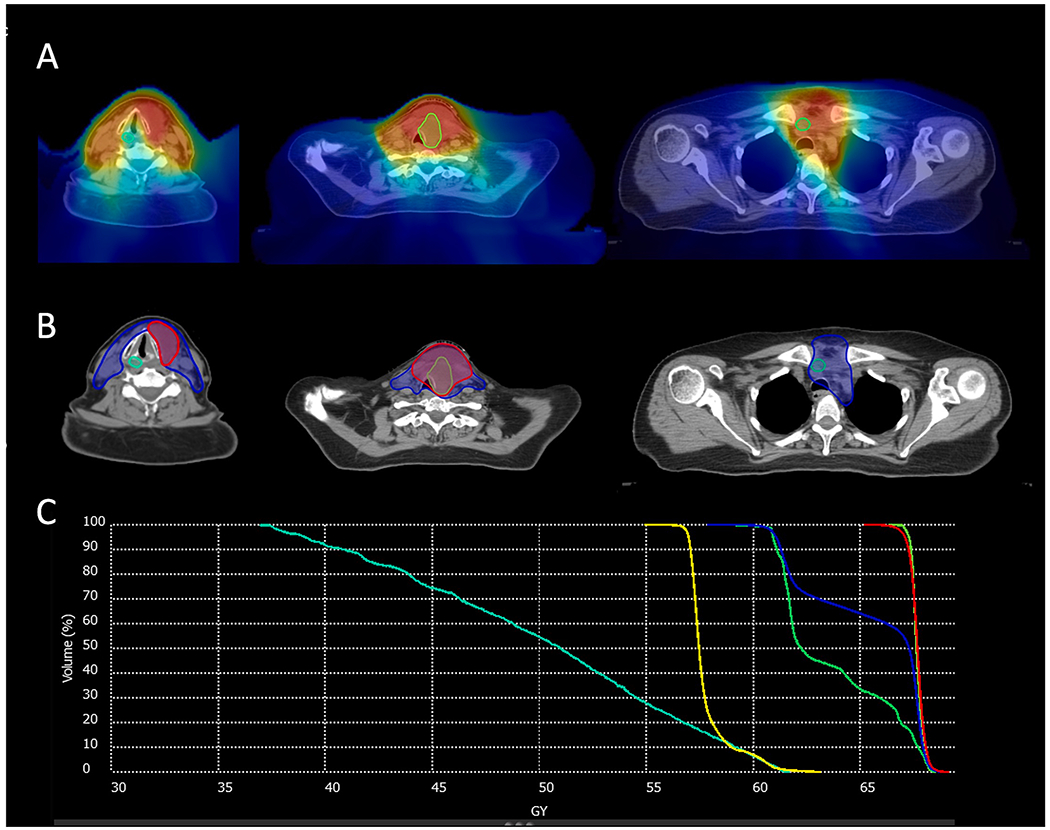
Workflow depicting identification and scoring of failures. A. Recurrent gross tumor volumes (rGTV, green) are contoured on diagnostic imaging, B. Deformable registration of original planning CT and diagnostic imaging is performed, C. rGTVs are co-registered to the original treatment plan allowing for spatial and dosimetric analysis at each location of recurrence (D). Red represents the original treatment plan high dose CTV, blue, intermediate dose CTV, and yellow, elective dose CTV. In this example, an illustration of a Type A, Type C, and Type E failure in the same patient is shown. (For interpretation of the references to colour in this figure legend, the reader is referred to the web version of this article.)

Post-treatment/follow-up diagnostic CT neck images and reports were reviewed to identify patients with and locations of LRP after IMRT. LRP after IMRT was defined using relatively strict criteria as any increase in size of treated disease or development of new disease in the neck or upper mediastinum. IMRT plan data were retrieved for patients with evidence of LRP. Recurrent GTVs (rGTVs) were individually and manually contoured on diagnostic CTs and co-registered with initial planning CTs using deformable image registration (Velocity AI, Fig. 1). Deformed rGTVs were spatially conceptualized using a validated centroid-based technique [14] where the center location of each rGTV was considered as the origin of each treatment failure.

### Patterns of failure classification

To determine dose differences between failure categories, dose volume histogram (DVH) plots were constructed to evaluate dose-threshold parameters associated with rGTV classification. rGTVs and patterns of LRP were classified based on established spatial and dosimetric criteria defined as follows:

*Type A: Central high dose failure;* centroid of mapped failure originated in a high-dose TV AND dose to 95 % of the failure volume (fd95%) was > 95 % of the dose prescribed to the TV.

*Type B: Peripheral high dose failure;* centroid of mapped failure originated in a high-dose TV AND fd95% was < 95 % of the dose prescribed to the TV.

*Type C: Central elective dose failure;* centroid of mapped failure originated in an intermediate or low-dose TV AND fd95% was > 95 % of the dose prescribed to the TV.

*Type D: Peripheral elective dose failure;* centroid of mapped failure originated in an intermediate or low-dose TV AND fd95% was < 95 % of the dose prescribed to the TV.

*Type E: Extraneous dose failure;* centroid of mapped failure did not originate in any TV but located in the neck or upper mediastinum.

An example of image registration, rGTV delineation, and dosimetric analysis is shown in Fig. 1.

### Statistical analysis

Statistical analysis was performed in R version 4.0.4 (2021-02-15). To evaluate the potential impact on patient outcomes and benefit of durable neck control after definitive-dose IMRT and given the high competing risk for distant progression, freedom from LRP (FFLRP) was the endpoint of interest selected to assess outcome after IMRT [15]. LRP events were defined at the time of first LRP. Patients were censored at time of death or at time or last follow-up if they were alive with no evidence of LRP. Survival was estimated using the Kaplan-Meier method. Differences between populations were compared by log-rank testing.

## Results

Patient and treatment characteristics are summarized in Table 1. Forty patients were included, of which 21 (53 %) were stage IVB and 19 (47 %) were stage IVC. Twenty-three (58 %) patients had undergone a macroscopically incomplete resection (R2), of which 22 (96 %) were performed at an outside institution and were either aborted when unresectable disease was encountered or were performed as a diagnostic procedure. In all these cases, the persistence of gross residual disease was verified on postoperative diagnostic imaging and reports.

IMRT had been planned using Pinnacle or RayStation treatment planning systems and delivered with daily image-guidance and 6MV photons. Dose and fractionation schemes were determined by the treating physician and all patients received a dose ≥45 Gy. 29 patients (81 %) were treated with multiple dose levels using a simultaneous integrated boost technique. Twenty (50 %) patients received accelerated hyperfractionation (2 fractions per day, for a total of 10 fractions per week; median fraction size 1.5 Gy; BED10 85.8 assuming 3 Gy per day), 16 (40 %) with standard fractionation (5 fractions per week; median fraction size 2 Gy, BED10 79.2), and 4 (10 %) with accelerated fractionation (6 fractions per week, BID once per week; median fraction size 2 Gy, BED10 80.2 assuming on a weekly basis: 2 Gy per day for days 1–4, 4 Gy on day 5). Nine out of the 16 patients (56.3 %) undergoing standard fractionation did so as part of a prospective protocol [16,17]. All patients received concurrent chemotherapy. Median time from IMRT start to completion was 32 days (range 23–53 days).

TV delineation was determined by the treating physician and reviewed prior to treatment planning by at least two additional head and neck-specialized radiation oncologists. In general, neck levels III, IV and VI were uniformly included in the TVs. Regarding inclusion of additional lateral neck levels in TVs, neck levels were uniformly included if involved at time of RT or prior to surgery (if preformed) and the next echelon adjacent to gross disease considered for inclusion in elective TVs. For example, for a patient with lateral neck disease involving level III, then level II would be considered for inclusion in elective TVs. Level V would be generally included in elective TVs for a patient with lateral neck disease in any level. Additional example cases are included in Supplemental Fig. 1.

Thirteen (33 %) patients had bilateral lateral neck involvement and 27 (67 %) had unilateral or no lateral neck involvement at the time of IMRT. The TV coverage for these groups by anatomic/neck level and laterality and anatomic location of LRP are shown in Table 2. The retropharyngeal nodal stations were not electively treated and were only included in 2 patients, both of whom had clinical evidence of retropharyngeal nodal involvement. 6 of 27 (22 %) patients with unilateral or no lateral neck involvement were selected for TVs limited to the unilateral neck and did not receive RT to the uninvolved/contralateral lateral neck. Of these, 3 (50 %) patients treated with omission of the contralateral lateral neck also had omission of the contralateral central compartment. Only uninvolved neck levels III-IV were included in the elective dose CTV in the remaining 21 patients (78 %).

Systemic therapy after IMRT is shown in Table 1. Thirty-nine patients had known BRAF mutation status and of these, 10 (25.6 %) were BRAFV600E mutated, and 6 of these would subsequently receive BRAF-directed systemic therapy. Of the remaining 4 patients with BRAFV600E mutation that did not receive BRAF-directed systemic therapy, 3 died within 4 months of IMRT completion and 1 received immunotherapy at the time of distant progression (treated in 2014).

**Fig. 2.**
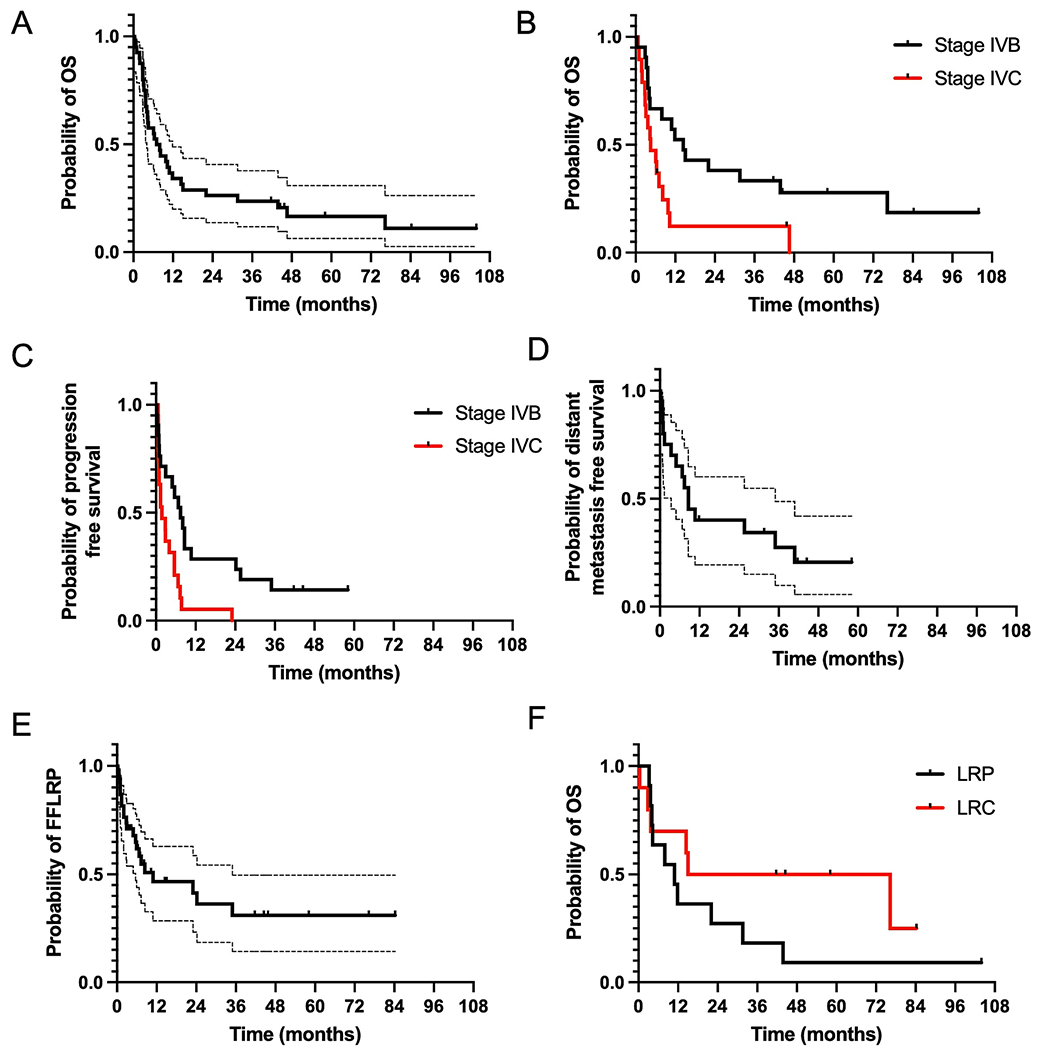
Patient outcomes after IMRT for unresected or incompletely resected ATC. (A) Median OS from completion of radiation therapy was 7.1 months: 14.4 months for patients with Stage IVB disease at the time of IMRT, and 4.5 months for patients with Stage IVC disease at the time of IMRT (B, p = 0.01). Median time to any progression was 7.4 months for patients with Stage IVB disease and 1.8 months for patients with Stage IVC disease (C, p = 0.003). (D) In patients with Stage IVB disease, time to distant metastasis was 8.6 months. (E) Median FFLRP was 10.9 months. (F) In patients with Stage IVB disease, median OS was 10.9 months for patients with locoregional progression versus 45.6 months for patients with locoregional control (p = 0.20).

Median follow-up after IMRT was 6.8 m from the end of IMRT for all patients. Six patients (15 %) were alive at the time of last follow-up. Median OS was 7.1 months from the end of IMRT (14.4 months for stage IVB, 4.5 for IVC, p = 0.01, Fig. 2 A,B). Median time to any disease progression from the end of IMRT was 7.4 months for stage IVB and 1.8 months for IVC (p = 0.003), Fig. 2C.

Thirty-one patients (78 %) developed distant progression after IMRT at a median of 5.5 months (IQR 1.0–25.6), 15/21 (71 %) with newly metastatic disease and 16/19 (84 %) with progression of existing metastatic disease. In patients with Stage IVB disease, median time to distant metastasis was 8.6 months (Fig. 2D). Five patients (12.5 %) required airway intervention (tracheotomy or tracheal stent) and 16 (40 %) a feeding tube during or after IMRT.

There were 9 patients with extended OS (≥36 months [range 41.6–104 months]) which are characterized in Table 3. Seven of these had Stage IVB disease and 2 Stage IVC (both with multiple FDG-avid lung nodules on presentation). All 9 had LRC for at least 23 months from the end of IMRT, and 6 (66.7 %) had LRC at last follow-up or death; of these, all had received ≥ 66 Gy, 4 had received AF, and 4 had no additional systemic therapy after IMRT. Median iGTV at time of IMRT in these longer-term surviving patients was 33.6 cc (1.1–181.7) versus 111.45 cc (4.2–479.2) for patients surviving less than 36 months (p = 0.03 nonparametric Mann-Whitney). Three patients had overall disease control following IMRT and concurrent chemotherapy alone (i.e. without adjuvant or additional systemic therapies). Of these, 1 had small volume residual disease (1.1 cc) after an initial R2 resection, indicative of a near gross total resection, and another had relatively small residual disease (<10 cc). Three of these longer-term survivors had BRAF V600E mutations and received BRAF-directed therapy.

Twenty-one patients (52.5 %) developed LRP at a median of 10.9 months (IQR 2.8-not reached) from completion of IMRT (Table 4, Fig. 2E). Six (28 %) patients had LRP as a first site of recurrence while 13 (62 %) had synchronous distant progression, and 2 (10 %) had distant progression prior to LRP. Fifteen out of 21 patients (71 %) who developed LRP died from progression of loco-regional disease. FFLRP at 3- and 12-months was 71 % (95 %CI 58–87 %) and 46.6 % (95 %CI 32–68 %). For patients who received ≥ 66 Gy (n = 35), FFLRP at 3- and 12-months was 68 % (95 %CI 54–85 %) and 44 % (95 %CI 28–67 %).

Eleven of 21 (52.4 %) patients with Stage IVB disease developed LRP. Of these, 8 (73 %) developed distant metastasis concurrently with or before LRP. In patients with Stage IVB disease, median OS was 10.9 months for patients with LRP versus 45.6 months for patients without (p = 0.20, Fig. 2F).

Seven patients (33 %) received systemic therapy prior to LRP either after IMRT or for distant progression (Table 4). FFLRP for patients who received adjuvant systemic therapy prior to LRP was 6.7 months compared to 2.0 months for those who did not receive systemic therapy after RT (p = 0.04). Median OS for patients who received systemic therapy prior to LRP was 43.8 months compared to 4.0 months for those who did not (p = 0.027).

On univariate analysis, there was no correlation between patient ECOG performance status, stage, iGTV, surgical resection versus biopsy only, or RT characteristics including dose, fractionation, or number of dose levels and FFLRP.

**Fig. 3.**
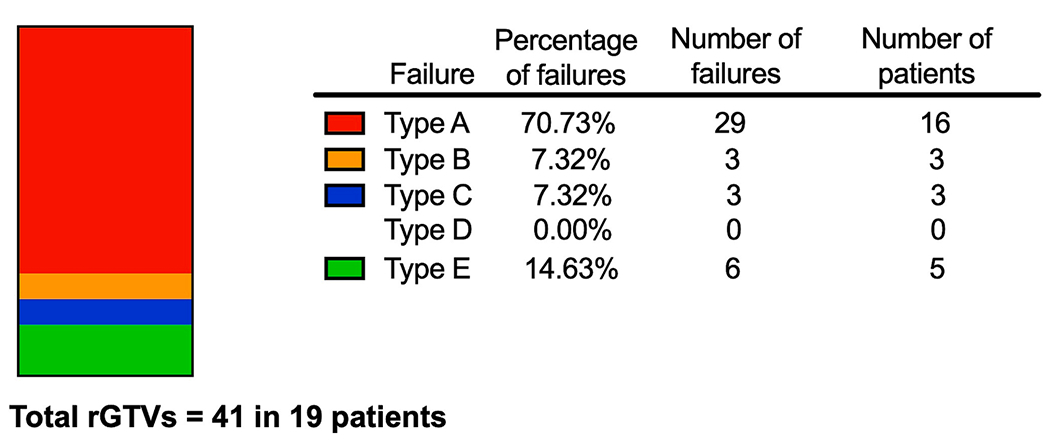
Recurrent gross tumor volumes (rGTV) categorized by spatial and dosimetric criteria. The majority of failures occurred in the high dose TV- Type A and B.

IMRT treatment plans were unable to be recovered (due to corrupted archived plan data) for 2 out of 21 patients with LRP. Overall, for the 19 evaluable treatment plans, 41 rGTVs were identified as most patients had more than one site of locally recurrent or progressive disease (n = 12, 63 %). Most failures occurred within the high dose CTV- Type A (n = 29, 71 %) and Type B (n = 3, 7 %) and most patients with LRP had at least one high dose failure (n = 17, 89 %). (Fig. 3). Three recurrences in 3 patients (3 Type C, 7 %) occurred in the elective dose CTV. The remaining 6 recurrences in 5 patients occurred in extraneous dose regions (15 %, Type E). In 7 patients who presented with a single failure site, there was 1 Type C failure and the remaining 6 were Type A. There was only 1 patient with an isolated Type E extraneous dose failures which occurred 35 months after completion of IMRT in an untreated parotid gland. df95% to these failure volumes were 8 Gy and <2 Gy respectively. This patient was able to undergo a salvage parotidectomy followed by post-operative IMRT to the previously untreated parotid bed.

Fourteen patients with unilateral or no lateral neck involvement had LRP at 26 sites. There were 3 Type E extraneous dose failures: 2 in the mediastinum (thoracic levels 3A and 6) and 1 *retro*-tracheal, and 3 type C elective dose failures: 1 posterior pharyngeal at the level of the hyoid, 1 level II in the involved neck, and 1 in the superior mediastinum (thoracic level 1). The remaining 20 failures were within the high dose CTV and classified as Type A or B (3 nodal failures where there was iGTV at the time of IMRT, 17 central). 13 (93 %) patients had at least 1 Type A failure.

Seven patients with bilateral lateral neck involvement had LRP at 15 sites. There were 3 Type E failures: 2 within the parotid (patient history described above) and 1 in level II neck that was not included in the elective TV. There were no Type C elective dose failures. The remaining 12 failures were within the high dose CTV (2 nodal failures where there was iGTV at the time of IMRT, 10 central).

Median prescription dose to the high dose CTV for patients with a Type A/B failure was 66 Gy (range 45–68). Median FFLRP was 22.9 months for patients without a Type A/B failure (n = 2) compared to 2.9 months for those with type A/B failures (n = 17, p = 0.06). Median OS was 33.0 months for patients without a type A/B failure compared to 4.5 months for those with type A/B failures (p = 0.41).

## Discussion

This study examines the patterns of loco-regional failure using spatial and dosimetric criteria and typology for patients with unresected or incompletely resected ATC treated with definitive-dose RT. In patients with localized ATC, tri-modal therapy beginning with upfront surgery is recommended when feasible by both the National Comprehensive Cancer network guidelines and American Thyroid Association recommendations [18,19]. Adjuvant RT following surgery has been shown to improve FFLRP and OS in multiple retrospective studies [2,5,20]. However, for patients with unresectable disease, or gross residual disease after attempted resection and no targetable mutations, the benefit of definitive-dose RT as a means of durable LRC and palliation is less defined. Due to the aggressive behavior of ATC, these patients are at highest risk for life-threatening morbidity associated with often rapid LRP which may compromise critical structures near the thyroid including the esophagus, larynx, and trachea. Difficulties in standardizing and analyzing the role of RT in the treatment of patients with gross disease are multifactorial and include the rarity of ATC and significant variations in treatment approaches not only by institution but also evolution of treatment paradigms over time [8].

To benchmark RT-associated LRC outcomes, we have adopted a standardized method, previously validated in other head and neck disease sites, to systematically categorize loco-regional disease recurrence/progression and quantitatively evaluate each failure by DVH distributions [21,22]. While traditional reports of loco-regional failure following RT are typically classified as in-field, marginal, or out-of-field based on the degree of overlap between sites of failure and RT TVs either through visual comparison or rigid image registration, the deformable image registration/centroid based approach employed for the current study allows for evaluation of the spatial components of failure in the context of precise dosimetric analyses [14]. This provides significant advantages over standard methods as it [1] facilitates localization and optimized spatial accuracy of recurrent disease with respect to the original treatment fields, and [2] increases confidence in actual dose versus assumption of dose delivered to the failure volume. These are essential considerations when classifying treatment failures after head and neck IMRT, which typically involve complex TVs, multiple dose levels, and steep dose gradients.

Using this methodology, we found that Type A failures, that is failures that represent iGTV resistance to the maximum prescribed dose, predominated in patients with LRP, despite aggressive treatment approaches including the routine use of concurrent chemotherapy and commonly altered fractionation. When they occurred, high dose failures occurred rapidly after IMRT completion and trended towards worse OS compared to those with failures only in elective dose or extraneous dose areas. Indeed, given the high competing risk for development or progression of distant disease and rapidity of high dose failures, adaptive processes during RT to identify poor responders are warranted. Most recently at our institution, patients undergoing definitive-dose IMRT routinely undergo restaging imaging on a case-by-case basis after receipt of ~40 Gy to identify those with rapidly progressive or high-volume disease outside the treatment fields and allow the opportunity for adjustment or discontinuation of the RT treatment course or overall treatment intent to potentially avoid unnecessary acute RT-related toxicity for patients who are unlikely to benefit.

While just over half of the patients in this cohort eventually experienced LRP, distant sites were the predominant location of first progression/failure highlighting the aggressive nature and metastatic potential of ATC. Even in patients with localized disease at diagnosis in our cohort, LRP after IMRT prior to distant failure was rare. However, we did identify a trend towards improved OS in those with LRC compared to those with LRP in patients with upfront localized disease (Stage IVB). The benefit of local therapy and importance of LRC must be considered in the context of improving systemic therapies [23]. On subanalysis of our cohort of patients with LRP, receipt of systemic therapy including targeted therapy prior to LRP was associated with trends towards extension in time to LRP and OS. This complements a previous report of survival outcomes in 426 patients with ATC treated over a 20-year time span at our institution which revealed a significant improvement in OS in patients treated after 2017 [8]. Investigators identified factors contributing to improved OS including the use of targeted therapy, immunotherapy, and surgery after neoadjuvant targeted therapy. Thus, it is unclear as to if the trend towards improved OS in patients without LRP can be attributed to solely local therapy, more effective systemic therapy or both. Nevertheless, with an aggressive treatment approach of prescribed dose of ≥66 Gy with concurrent chemotherapy, durable LRC was achieved for ~47 % of this patient cohort at 12 months after completion of IMRT. Likewise, the relatively low rates of airway interventions and feeding tube placement during/after RT highlights the potential durable palliative benefit of definitive-dose RT with concurrent chemotherapy.

Our findings expand considerably on previous efforts to describe patterns of loco-regional progression in patients receiving RT for ATC. In a small series of 5 patients with ATC, Vulpe et al. reported a LRP rate of 60 % at 1 year and 100 % at 2 years. Seven recurrent volumes were identified and were evenly split between infield, marginal, and out-of-field locations. Study authors concluded the pattern of spread could not be attributed to an orderly progression, and coverage of all sites of failure would lead to unacceptable toxicities [24]. Indeed, these coupled with our findings of the patterns of LRP after IMRT highlight the importance of further investigation of strategies to overcome disease radio-resistance for treatment of ATC, potentially with novel radiation sensitizers or iso-toxic dose escalation (e.g. intralesional boost or linear energy transfer-optimized proton/particle therapy).

In a more recent study, investigators retrospectively reviewed outcomes for 47 patients with ATC treated with definitive or adjuvant RT [25]. Of note, only 8 patients with unresectable or incompletely resected disease (R2 resection) were included in this report. In the combined group of patients, 29.8 % experienced infield recurrence within the thyroid gland or postoperative tumor bed, 8.5 % had marginal failures with the majority occurring in the mediastinum, and 4.2 % had out-of-field failures. As per the reporting institution’s practice, all patients received elective nodal neck RT to bilateral levels II-IV and VI as well as the majority also receiving treatment to level V and mediastinal levels 1–6. On analysis of LRP in the combined cohort, investigators concluded that aggressive, comprehensive RT coverage should include bilateral levels II-V, VI, and the mediastinum to the level of the carina. In the cooperative group trial, RTOG0912, examining the use of IMRT with concurrent paclitaxel with or without pazopanib for patients with ATC, the protocol required elective dose coverage of neck levels II-VI bilaterally and the upper mediastinum down to the level of the carina with coverage of level I and retropharyngeal nodes at the discretion of the treating physician [16,17,26]. Results of RTOG0912 with a median follow up of 2.9 years have recently been reported showing 1-year LRF rates of 28.6 % in the experimental arm and 33.6 % in the control arm and 1-year OS of 36.1 % in the experimental arm and 23.2 % in the control arm [16]. These are not directly comparable to our results given the absence of information of the completeness of surgery, burden of residual disease at the start of RT or central review of loco-regional imaging to determine LRP using the relatively strict criteria applied in our study.

In contrast to these practices, we employed more limited RT TVs with the majority of patients receiving RT to levels VI and the mediastinum to the level of the aortic arch, at-risk levels III/IV, and omitting coverage or limiting inclusion of uninvolved lateral neck levels on a case-by-case basis, all in effort to mitigate treatment-related toxicity. Despite employing these more selective TVs, isolated Type E failures in the extraneous dose regions occurred in only 1 patient who was the latest to recur after completion of RT and recurred in a site (parotid) that would not have been routinely included using more comprehensive RT coverage and could reasonably be considered a site of distant failure. This patient was able to receive successful salvage therapy and had extended OS compared to that observed for patients with Type A, central high dose failures. Furthermore, using this approach of more selective lateral neck coverage, there was only 1 Type E failure within the level II nodal station where coverage was omitted. This patient had an additional synchronous high-dose failure in the central neck. There were no Type E failures in the uninvolved neck in patients with no or only unilateral lateral neck involvement prior to RT. Given the primacy of controlling gross disease for durable palliative benefit and the risk of high dose failures, these data suggest a more selective use of elective TVs may be employed in appropriately selected patients without excess risk of worsened outcomes due to failure in untreated regional sites. These may include patients without lateral neck disease or well-lateralized disease for example.

Given the retrospective nature and limited power of this study, we acknowledge the limitations in this data. Importantly, the influence of having biopsy only versus receiving incomplete surgery on patient outcomes is subject to substantial selection biases. For example, those with disease deemed amenable to an attempt at surgical resection prior to presenting to our institution were likely to have an overall lesser disease burden or better performance status compared to those with biopsy only. Furthermore, some surgeries were likely performed for diagnostic purposes only. Additionally, the possibility cannot be ruled out that patients in this cohort judged to have had small volume gross residual disease as determined by review of postoperative imaging and reports could be an overestimation (i.e. ‘Overcall”) of actual benign postoperative change after gross total resection. Likewise, this study was limited to patients who were selectively treated with definitive-dose RT and thus unfit patients or those with overwhelming disease burden, whether loco-regional or distant, and those who received lower/palliative dose RT are not represented. Additionally, patients in our cohort with very aggressive local disease may have rapidly succumbed to disease or others may have experienced significant decline in performance status (whether from disease or treatment-related toxicity) potentially precluding the initiation of systemic therapy after RT, thus contributing to difference in outcomes observed. Similarly, in our standardized assessment of the available data, we only reviewed patients who received neck RT at our institution, which treats a high volume of patients with ATC and where treatment planning is rigorously evaluated through multidisciplinary quality-assurance which may impact generalizability of our reported outcomes to other clinical cohorts. Furthermore, inherent to the rapidity of ATC recurrence and progression, LRP dates are approximations of time to first treatment failures as detected on imaging. Though patients are frequently followed, interval growth or seeding of tumors between follow-up intervals cannot be excluded. Selection bias among patients considered suitable for higher RT doses, altered fractionation or systemic therapies are additional possible limitations of the current study. Most importantly, this study reports on patients treated and outcomes during the emergence and optimization of therapies in the targeted therapy era. These findings would be most applicable in current clinical practice for patients with localized but unresectable disease, and without targetable mutations, as patients with BRAF-mutated ATC would typically undergo neoadjuvant systemic therapies/trials [10].

Ongoing investigational efforts to improve outcomes in patients with ATC seek to optimize both local–regional and distant disease control. These include the use of neoadjuvant targeted/immunotherapy in those with actionable mutations, such as BRAF V600E and BRAF-directed therapy, followed by consolidative local therapy, generally surgery with or without postoperative radiation (NCT04675710, NCT04739566). The practice of neoadjuvant dabrafenib/trametinib has already been included in the ATA and NCCN guidelines [18,19]. Additionally, the role of immunotherapy after RT for patients with unresectable disease is currently under investigation (IMPAACT-NCT05059470); however, early an early pilot study of concurrent chemoradiation therapy combined with immunotherapy did not appear to show improvement in survival outcomes compared to historical controls [27]. Other strategies to enhance radiosensitization in ATC tumors are desperately needed. However, currently, for fit patients presenting with unresectable ATC, BRAF wild-type, without or only limited volume distant disease, the treatment paradigm of upfront definitive-dose IMRT with concurrent systemic therapy with the TV delineation strategies described herein with goals of durable LRC remain applicable to modern practice.

## Supplementary Material

Supplementary Data

## Figures and Tables

**Table 1:** Patient and treatment characteristics.

	Overall (N = 40)
**Age at time of RT (y)**	
Mean (SD)	65.2 (8.77)
Median [Min, Max]	66.0 [45.0, 83.0]
**ECOG performance status**	
0	16 (40.0 %)
1	19 (47.5 %)
2	4 (10.0 %)
3	1 (2.5 %)
**AJCC Staging**	
IVB	21 (52.5 %)
IVC	19 (47.5 %)
**Surgical extent**	
Biopsy	17 (42.5 %)
R2 resection	23 (57.5 %)
**Residual gross tumor volume (cc) at time of IMRT**	
Mean (SD)	132 (137)
Median [Min, Max]	88.4 [1.10, 479]
**Days from surgery/biopsy to IMRT start**	
Mean (SD)	30.2 (13.9)
Median [Min, Max]	28.0 [4.00, 59.0]
**IMRT Dose, Gy**	
Mean (SD)	65 (4.48)
Median [Min, Max]	66 [45, 70]
**IMRT Fractionation**	
BID	20 (50.0 %)
ACC	4 (10.0 %)
SF	16 (40.0 %)
**Number of dose levels**	
Mean (SD)	2.40 (0.871)
Median [Min, Max]	2.00 [1.00, 4.00]
**High dose CTV volume (cc)**	
Mean (SD)	198 (201)
Median [Min, Max]	145 [10.4, 1140]
**Concurrent chemotherapy**	
Carboplatin/paclitaxel	26 (65.0 %)
Cisplatin	4 (10.0 %)
Other (RTOG-0912)	10 (25.0 %)
**Systemic therapy after IMRT**	26 (65 %)
Chemotherapy	17 (42.5 %)
Immunotherapy	14 (35 %)
BRAF-directed therapy	6 (15 %)
Other targeted therapy*	14 (35 %)

Min- minimum; Max- maximum; SD- standard deviation; IMRT- intensity modulated radiation therapy; CTV- clinical target volume; BID- twice daily fractionation; SF- standard fractionation; ACC- accelerated fractionation, 6 fractions per week.

* Other targeted therapies include levatinib, everolimus, pazopanib, investigational AKT inhibitor, bevacizumab, sorafenib, bortezomib, cobimetinib.

**Table 2:** Radiation target volume coverage and anatomic location of LRP.

Bilateral lateral neck involvement	Target volume coverage Number of patients		Anatomic location of LRP Number of patients	
N = 13	*Bilateral*	*Unilateral*		

RP	2 (15 %)			
IB	4 (31 %)	1 (8 %)		
II	8 (62 %)	4 (31 %)	2 (13 %), *1 within omitted level*	
III	13 (100 %)		1 (7 %)	
IV	13 (100 %)			
V	9 (69 %)	3 (23 %)		
Central compartment	13 (100 %)		10 (67 %)	
Superior mediastinum	13 (100 %)			
Other			2 (13 %)	
Unilateral/no lateral neck involvement	Target volume coverage Number of patients		Anatomic location of LRP Number of patients	
N = 27	*ipsilateral*	*contralateral*	*ipsilateral*	*contralateral*

RP	0 (0 %)	0 (0 %)		
IB	5 (19 %)	0 (0 %)		
II	17 (63 %)	0 (0 %)	2 (8 %)	
III	27 (100 %)	21 (78 %)	1 (4 %)	
rv	27 (100 %)	21 (78 %)		
V	21 (78 %)	0 (0 %)		
Central compartment	27 (100 %)		17 (65 %)	
Superior mediastinum	27 (100 %)		4 (15 %)	
Other			2 (8 %)	

Abbreviations: LRP- loco-regional progression; RP- retropharyngeal.

**Table 3:** Patient and treatment characteristics for patients with survival ≥ 36 months from end of IMRT.

Age (y)	ECOG	Stage	Extent of surgery	iGTV (cc)	iGTV location(s)	Dose (Gy)/Fractionation	Concurrent chemotherapy	Time to LRP (m)	Time to distant progression (m)	Followup (m)	Vital/Disease Status	BRAF status	Post-IMRT therapies received*
69	0	IVB	R2	1.1	Unifocal: pretracheal	66/BID	cisplatin	–	–	44.5	Alive/NED	WT	none
69	1	IVB	R2	3.0	Multifocal: R thyroid bed, R neck	69.3/SF	carbo/paclitaxel	–	25.6	76.3	Dead	WT	lung SBRT
70	0	IVB	R2	7.1	Multifocal: R thyroid bed	66/BID	carbo/paclitaxel	–	–	41.7	Alive/NED	WT	none
66	0	IVC	R2	39.5	Unifocal: L thyroid bed	66/BID	cisplatin	–	7.7	45.8	Alive/with disease	WT	atezolizumab, cobimetinib, lung SBRT
70	0	IVB	R2	157.2	Unifocal: L thyroid	66/BID	carbo/paclitaxel	–	–	58.1	Alive/NED	WT	none
68	0	IVB	R2	181.7	Multifocal: R thyroid bed, R neck	66/SF	other	–	8.6	84.3	Alive/with disease in remission (currently on therapy)	V600E mutation	chest wall skin metastatectomy and postoperative RT, dabrafenib, trametinib, pembrolizumab
63	1	IVB	R2	23.8	Multifocal; R thyroid, R neck, tumor venous thrombus	68/ACC	carbo/paclitaxel	24.2	40.8	104.0	Alive/with disease in remission (currently off therapy)	WT	carbo/paclitaxel, lenvatinib, ipilimumab, lung SBRT, pembrolizumab, everolimus
68	0	IVB	R2	33.6	Multifocal: R thyroid, B neck	66/SF	other	35.0**	35.0	43.8	Dead	V600E mutation	dabrafenib, trametinib, pembrolizumab, everolimus
56	1	IVC	Biopsy only	96.8	Unifocal: L thyroid	60/BID***	carbo/paclitaxel	23.0	–	46.6	Dead	V600E mutation	dabrafenib, trametinib, pembrolizumab, lenvatinib

BID: twice daily fractionation; ACC: accelerated fractionation (6 fractions/week); SF: standard fractionation; carbo = carboplatin; other: paclitaxel + pazopanib or placebo; WT = wildtype.

* Therapies listed do not represent indication, sequence or combinations used.

** LRP in parotid gland (see text), no other site of LRP.

*** patient elected to discontinue RT at 60 of a planned 66 Gy.

**Table 4:** Patient, treatment and failure characteristics for patients with loco-regional progression after IMRT (n = 21).

Age (y)	ECOG	Stage	Neck involvement at presentation	Extent of surgery	iGTV (cc)	Dose (Gy)	Dose/fraction (Gy)	Fractionation	Concurrent chemo	Systemic therapy prior to LRP	Time to LRP from end of RT (m)	OS (m)	Type A	Type B	Type C	Type D	Type E
45	1	IVB	none	R2	124.6	66	1.5	BID	carbo/paclitaxel	none	0.2	3.2	3-C				
55	2	IVC	bilateral	R2	105.5	66	1.5	BID	carbo/paclitaxel	none	0.6	1.0		1-C			1-N
71	1	IVC	bilateral	R2	111.5	66	1.5	BID	carbo/paclitaxel	carbo/paclitaxel	0.9	4.5	1-C				
56	0	IVC	unilateral	Biopsy	455.9	66	2	SF	other	none	0.9	1.8	1-C				
64	1	IVB	unilateral	R2	80.0	66	1.5	BID	carbo/paclitaxel	none	1.0	4.0	2-C1-N				1-M
51	0	IVC	unilateral	R2	10.1	66	1.5	BID	carbo/paclitaxel	none	1.3	3.7	Typology not analyzable				
66	2	IVC	unilateral	R2	137.7	66	1.5	BID	carbo/paclitaxel	none	1.3	2.0	1-C				
74	1	IVC	unilateral	Biopsy		66	2	SF	carbo/paclitaxel	none	2.0	2.8	Typology not analyzable				
49	0	IVB	none	R2	42.8	66	2	SF	other	none	2.0	3.6	2-C				
61	1	IVC	unilateral	Biopsy	172.0	66	1.5	BID	carbo/paclitaxel	carbo/paclitaxel	2.8	2.8	3-C	1-N			
70	1	IVB	bilateral	Biopsy	155.6	66	2	SF	carbo/paclitaxel	none	2.9	4.3	1-C				
82	1	IVB	none	R2	40.4	66	2	SF	other	none	4.9	10.9	1-C				
70	0	IVB	none	R2	35.7	66	1.5	BID	carbo/paclitaxel	none	5.6	31.6	1-C		1-M		1-RTr
58	0	IVC	bilateral	R2	60.2	66	1.5	BID	carbo/paclitaxel	lenvatinib	5.9	6.5 (alive)	2-N				
56	1	IVC	bilateral	Biopsy	417.3	45	1.5	BID	carbo/paclitaxel	BRAF-directedtherapy	6.7	7.1	3-C				
74	2	IVB	unilateral	Biopsy	474.7	66	2	SF	other	none	7.3	7.9	1-C	1-C			1-M
73	1	IVB	unilateral	R2	4.2	66	2	ACC	carbo/paclitaxel	none	8.4	11.8	1-C				
61	1	IVB	unilateral	R2	29.0	66	2.2	ACC	carbo/paclitaxel	none	10.9	22.1			1-RP		
56	1	IVC	unilateral	Biopsy	96.8	60	1.5	BID	carbo/paclitaxel	BRAF-directedtherapy	23.0	46.6	1-C		1-N		
63	1	IVB	bilateral	R2	23.8	68	2	ACC	carbo/paclitaxel	carbo/paclitaxel; lenvatinib	24.2	104 (alive)	4-C				
68	0	IVB	bilateral	R2	33.6	66	2	SF	other	BRAF-directedtherapy	35.0	43.8					2-P

IMRT- intensity modulated radiation therapy; C- Central; N- Nodal; M- Mediastinum; RTr- retrotracheal; RP- retropharyngeal; P-parotid; Gy- Gray; LRP- loco-regional progression; RT- radiation therapy; BID- 2 fractions per day, 10 fractions per week; SF- standard fractionation; (daily); ACC- 6 fractions per week; y- years; m- months; chemo- chemotherapy; OS- overall survival; carbo-carboplatin; other: paclitaxel + pazopanib or placebo.
