# Supplementary figures and images for: Patterns of loco-regional progression and patient outcomes after definitive-dose radiation therapy for anaplastic thyroid cancer
Source: Radiother Oncol. Author manuscript; Available in PMC 2025 Jan 10. (PMC11720968; doi:10.1016/j.radonc.2024.110602)

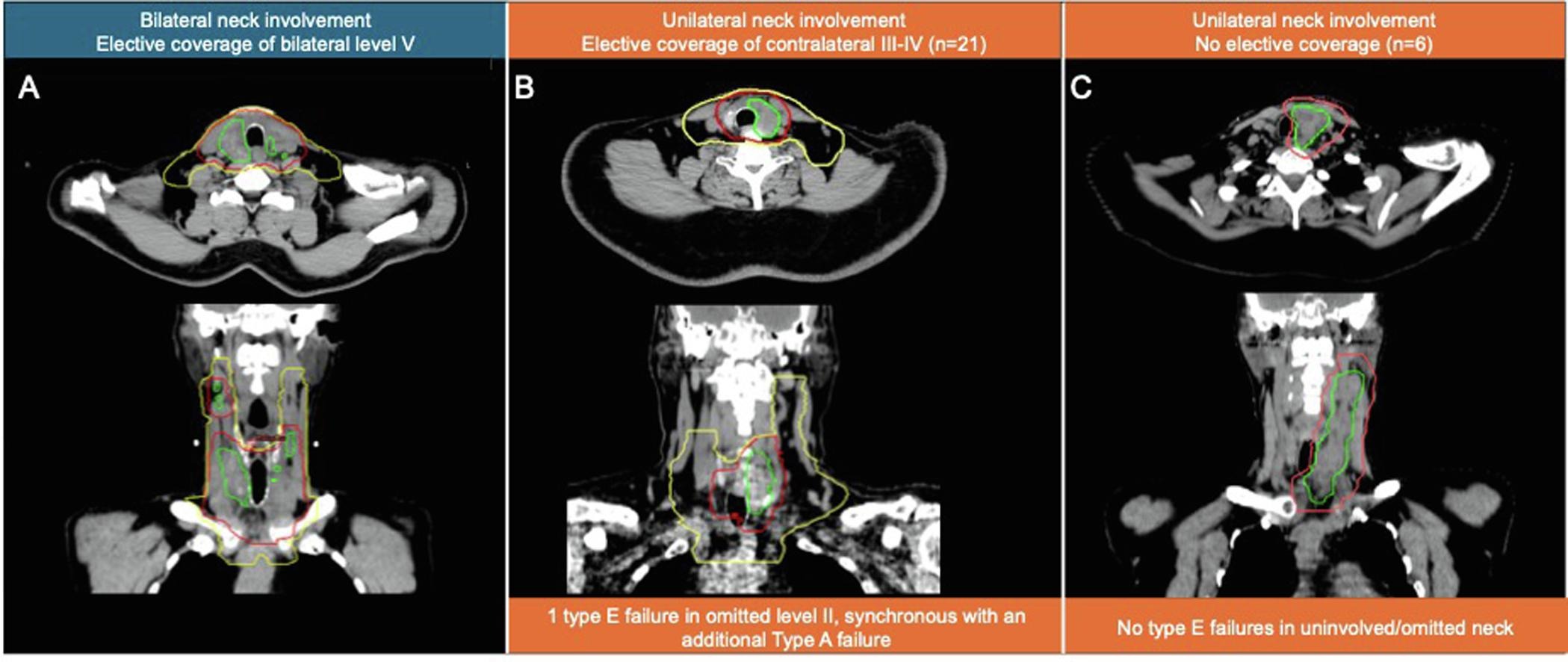

Supplement: Supplementary Data [file NIHMS2043170-supplement-Supplementary_Data.jpg]
